# Critical Review of Transcutaneous Vagus Nerve Stimulation: Challenges for Translation to Clinical Practice

**DOI:** 10.3389/fnins.2020.00284

**Published:** 2020-04-28

**Authors:** Jonathan Y. Y. Yap, Charlotte Keatch, Elisabeth Lambert, Will Woods, Paul R. Stoddart, Tatiana Kameneva

**Affiliations:** ^1^ARC Training Centre in Biodevices, Swinburne University of Technology, Hawthorn, VIC, Australia; ^2^Faculty of Science, Engineering and Technology, Swinburne University of Technology, Hawthorn, VIC, Australia; ^3^School of Health Sciences, Swinburne University of Technology, Hawthorn, VIC, Australia; ^4^Iverson Health Innovation Research Institute, Swinburne University of Technology, Hawthorn, VIC, Australia; ^5^Department of Biomedical Engineering, The University of Melbourne, Parkville, VIC, Australia

**Keywords:** vagus nerve, vagus nerve stimulation, transcutaneous, neuromodulation, neurostimulation

## Abstract

Several studies have illustrated that transcutaneous vagus nerve stimulation (tVNS) can elicit therapeutic effects that are similar to those produced by its invasive counterpart, vagus nerve stimulation (VNS). VNS is an FDA-approved therapy for the treatment of both depression and epilepsy, but it is limited to the management of more severe, intervention-resistant cases as a second or third-line treatment option due to perioperative risks involved with device implantation. In contrast, tVNS is a non-invasive technique that involves the application of electrical currents through surface electrodes at select locations, most commonly targeting the auricular branch of the vagus nerve (ABVN) and the cervical branch of the vagus nerve in the neck. Although it has been shown that tVNS elicits hypo- and hyperactivation in various regions of the brain associated with anxiety and mood regulation, the mechanism of action and influence of stimulation parameters on clinical outcomes remains predominantly hypothetical. Suppositions are largely based on correlations between the neurobiology of the vagus nerve and its effects on neural activity. However, tVNS has also been investigated for several other disorders, including tinnitus, migraine and pain, by targeting the vagus nerve at sites in both the ear and the neck. As most of the described methods differ in the parameters and protocols applied, there is currently no firm evidence on the optimal location for tVNS or the stimulation parameters that provide the greatest therapeutic effects for a specific condition. This review presents the current status of tVNS with a focus on stimulation parameters, stimulation sites, and available devices. For tVNS to reach its full potential as a non-invasive and clinically relevant therapy, it is imperative that systematic studies be undertaken to reveal the mechanism of action and optimal stimulation modalities.

## 1. Introduction

Vagus nerve stimulation (VNS) is an FDA-approved treatment for both pharmacoresistant depression and epilepsy and can produce clinically meaningful antidepressant and anti-seizure effects (Nemeroff et al., 2006; Johnson and Wilson, 2018). More than 100,000 VNS devices had been implanted in more than 70,000 patients globally by 2013 (Labiner and Ahern, 2007). The implantable device consists of an electrode, which is wrapped around the left vagus nerve, and an implantable unit, positioned below the collarbone and containing the battery and pulse generator.

Device implantation is predominantly performed on an outpatient basis under general anesthetic, but some patients may require overnight stay if extended observation is necessary. Despite being a minimally invasive procedure, the surgery is inherently risky due to the location of implantation, with electrode placement requiring dissection of the vagus nerve from the carotid artery. Potential adverse events arising from the surgical intervention include bradyarrhythmias during device placement, the development of peritracheal hematoma (due to surgical trauma), and other respiratory complications, including vocal cord dysfunction and dyspnea (due to nerve trauma). VNS can also cause changes to breathing patterns during sleep, resulting in an increase in the number of obstructive apneas and hypopneas (Marzec et al., 2003; Fahy, 2010), and can, albeit rarely, produce late-onset bradyarrhythmias and severe asystolia due to atrium-ventricular block (Iriarte et al., 2009). These potential adverse events limit the intervention's applicability to those who are resistant to conventional therapeutic strategies, and total device and procedural costs amount to around AU $50,000 (Lehtimäki et al., 2013), a price that is prohibitively high for many, as it is a non-subsidized treatment.

**Figure 1:**
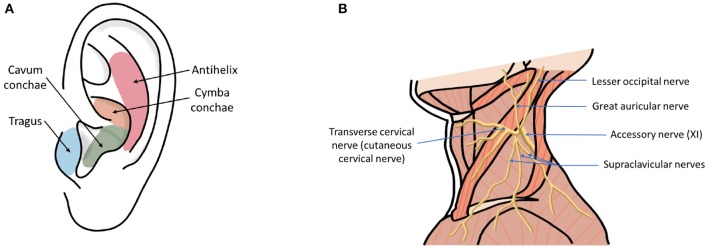
**(A)** Ear regions with innervation by the cutaneous auricular branch of the vagus nerve (ABVN). **(B)** Nerves in the neck region including cervical branch of the vagus nerve.

Transcutaneous vagus nerve stimulation (tVNS) is a method that has been developed to overcome these limitations, and the potential widespread accessibility of the technology adds to its appeal as a possible first-line treatment option. Anatomical studies of the ear suggest that the tragus, concha, and cymba concha are the places on the human body where there are cutaneous afferent vagus nerve distributions (Figure 1) (Peuker and Filler, 2002), and it is believed that stimulation of these afferent fibers should produce therapeutic effects that are similar to those of regular VNS (Hein et al., 2012; Rong et al., 2012; Stefan et al., 2012). Similarly non-invasive stimulation of the cervical branch of the vagus nerve has received popularity due to minimal side effects, low cost, and morbidity associated with the technique (Goadsby et al., 2014; Grazzi et al., 2014; Kinfe et al., 2015b). In this review, we refer to both auricular and cervical nerve stimulation as tVNS.

The potential of tVNS is not limited to the treatment of depression and epilepsy, with the technology being investigated for a variety of disorders including headache, tinnitus, atrial fibrillation, post-error slowing, prosocial behavior, associative memory, schizophrenia, and pain (Laqua et al., 2014; Hasan et al., 2015; Hyvärinen et al., 2015; Jacobs et al., 2015; Nesbitt et al., 2015; Sellaro et al., 2015a,b; Stavrakis et al., 2015).

Despite the breadth of research being undertaken, many questions remain regarding the most effective stimulation sites and parameters. As many of the described methods differ in the parameters and protocols applied, there is currently no firm evidence regarding the optimal location for stimulation to achieve the greatest clinical effects let alone an understanding of the neurophysiological mechanisms. Therefore, this critical review aims to explore the reported studies in tVNS with a view to promoting more systematic approaches that might help to translate the technique into mainstream clinical practice.

In comparison to tVNS, the invasive approach to VNS has been the subject of a number of recent reviews. For example, a review of functional neuroimaging studies in VNS confirmed that invasive stimulation causes changes in various brain regions and at different levels (Chae et al., 2003). A review of VNS with a focus on depression is presented in Müller et al. (2018). Recent advances in devices for VNS have been covered in Mertens et al. (2018). Similarly, applications and potential mechanisms of VNS have been discussed in some detail (Groves and Brown, 2005; Yuan and Silberstein, 2016a,b).

The few reviews that specifically focus on tVNS are very recent. A systematic review of the safety and tolerability of tVNS was presented in Redgrave et al. (2018), while two companion papers have focused on the physiological and engineering perspectives of tVNS (Kaniusas et al., 2019a,b). Whereas, Kaniusas et al. (2019a,b) outlined current research directions in auricular vagus nerve stimulation, this review takes a more critical approach and explores fundamental limitations of study design protocols that may lead to difficulties in translating current research into the clinic. We have also reviewed cervical vagus nerve stimulation in addition to auricular applications.

The review presented here focuses on a mechanistic understanding of tVNS, with a detailed description of stimulation parameters, sites of stimulation, and devices used in current research. We review current publications investigating the effect of electrode placement on auricular vagus nerve stimulation recruitment and corresponding neural activations, papers studying the effect of stimulation parameters (waveform, polarity, frequency, pulse width, duty cycle, and current), and manuscripts exploring the neurophysiological mechanisms of tVNS. We also consider whether tVNS can be used for closed-loop control of neural activity. We outline fundamental gaps in our understanding that need to be overcome in order to maximize efficacy, minimize risk, and thus support the successful translation of tVNS into mainstream clinical practice.

## 2. Transcutaneous Vagus Nerve Stimulation (tVNS)

### 2.1. Anatomical Considerations

**Figure 2:**
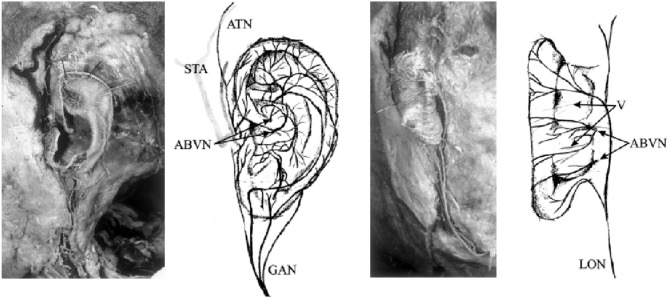
Innervation of the auricular branch of the vagus nerve (ABVN). GAN, great auricular nerve; ATN, auriculotemporal nerve; STA, superficial temporal artery; LON, lesser occipital nerve; V, vessels. Adapted from Peuker and Filler (2002) with permission.

Transcutaneous vagus nerve stimulation (tVNS) is based on the results of anatomical studies illustrating the path of the auricular branch of the vagus nerve (ABVN; Alderman's nerve; and Arnold's nerve), which originates from the superior ganglion of the vagus nerve from within the jugular foramen (Tekdemir et al., 1998), transversely passing through the facial canal, entering the small canal of the petrous bone, and emerging from the tympanomastoid fissure, proceeding to innervate the external acoustic meatus and auricle (Kiyokawa et al., 2014). As Peuker and Filler identify, the ABVN (Figure 2) is most prominently spread through the antihelix, tragus, cymba concha, and concha (Peuker and Filler, 2002). These are the places on the human body where there are cutaneous afferent vagus nerve distributions, and thus, as theoretically proposed by Ventureyra (2000), it is believed that direct stimulation of these nerve fibers should produce therapeutic effects similar to those of VNS. More recently, the original article by Peuker and Filler was the subject of some controversy due to different numbers being reported for tragus innervation by the ABVN in the main text and in the table (possibly a typing error) (Burger and Verkuil, 2018). Peuker and Filler (2002) later explained that the knowledge of auricular vagus nerve anatomy does not rest solely on this data, and other publications support the same findings (He et al., 2012).

**Figure 3:**
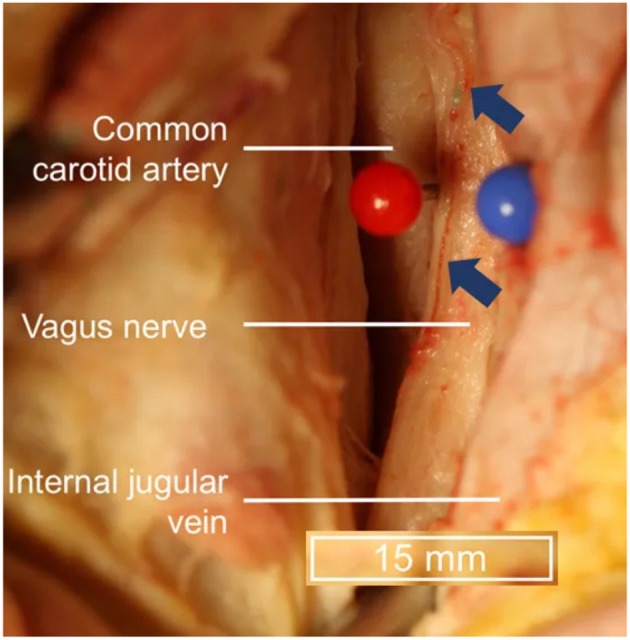
Topography of vagus nerve anatomy in the neck. Blue arrows indicate vessels external to the epineurium. Adapted from Hammer et al. (2018) with permission.

Transcutaneous cervical vagus nerve stimulation is another method that has been developed to non-invasively stimulate the vagus nerve with electrodes placed over the sternocleidomastoid muscle. This is a similar location to where the electrodes for VNS are positioned and is more reminiscent of Corning's initial approach. However, the vagus nerve's location within the carotid sheath (Figure 3), beneath the skin (2 mm), superficial fascia (3–6 mm), and sternocleidomastoid muscle (5–6 mm) (Seiden et al., 2013) can make selective transcutaneous stimulation of vagus nerve fibers difficult, with current product offerings most likely indiscriminately stimulating afferent and efferent fibers alike (Yuan and Silberstein, 2016b).

Conventionally, the left vagus nerve has mostly been selected as the preferred stimulation site due to safety concerns arising from observations during animal studies showing that right-sided VNS results in a greater degree of bradycardia (Yuan and Silberstein, 2016b). This is due to the asymmetric innervation of the heart, where the right vagus nerve predominantly innervates the sinoatrial (SA) node and the left predominantly innervates the atrioventricular (AV) node (Ardell and Randall, 1986). As such, right VNS in dog studies activated the cardiac motor efferents innervating the SA node, causing bradycardia through a reduction of depolarization rates and providing credence to the belief that right-sided VNS should not be attempted in clinical settings (Krahl, 2012). However, the anatomy of the cervical vagus trunk differs between dogs and humans, and the location around which the VNS stimulation electrodes are wrapped (in humans) does not include the superior or inferior cardiac branches, thereby diminishing the risk of significant cardiac adverse events (Krahl, 2012). Despite this, the FDA-approved labeling for VNS devices specifies that “the VNS Therapy System is indicated for use only in stimulating the left vagus nerve in the neck area inside the carotid sheath. The VNS Therapy System is indicated for use only in stimulating the left vagus nerve below where the superior and inferior cervical cardiac branches separate from the vagus nerve. The safety and efficacy of the VNS Therapy System have not been established for stimulation of the right vagus nerve or of any other nerve, muscle, or tissue” (Depression Physician's Manual, 2005).

While limiting treatments to the left side may be warranted for VNS, due to the potential to directly stimulate the cardiac motor efferents innervating the SA node, there are questions as to whether the application of these conventional reservations to tVNS is justified. The cardiac effects seen through ABVN stimulation are mediated through a neural pathway that involves the nucleus tractus solitarii (NTS); this activates the dorsal motor nucleus, which then delivers processed signals to the heart surface bilaterally via the efferent cervical vagus nerves. Therefore, unlike cervical VNS, tVNS circumvents the risk of directly and asymmetrically stimulating cardiac motor efferent fibers, thus causing adverse cardiac events (Chen et al., 2015). As such, simply disregarding the therapeutic potential of bilateral ABVN stimulation, based on conventional preconceptions and parallels drawn from VNS, may be premature and warrants further investigation. Additionally, bilateral ABVN stimulation has been shown to be safe in pilot studies investigating tVNS as a complementary therapy for pediatric epilepsy (He et al., 2013).

### 2.2. Nerve Fiber Types

The vagus and its branches consist of around 80% sensory afferent and 20% motor afferent fibers (Yu et al., 2008). Nerve fibers can be further classified into one of three groups based on their diameter: the A group (consisting of Aα Aβ, Aγ, and Aδ), B group, and C group. The different nerve fiber types have different diameters and myelination thicknesses (Table 1), which corresponds to different conduction velocities, with thicker myelination typically linked to faster conduction velocities or signal propagation (Fix and Brueckner, 2009).

**Table 1:** Classification of nerve fibers.

**Nerve fiber**	**Diameter**	**Myelination**	**Conduction** **velocity**	**Afferent or**	**Type**
**classification**	**(μm)**		**(m/s)**	**Efferent**	
Aα	13–20	Thick	80–120	Both	Sensory
					and Motor
Aβ	6–12	Medium	33–75	Both	Sensory
					and Motor
Aγ	5–8	Medium	4–24	Efferent	Motor
Aδ	1–5	Thin	3–30	Afferent	Sensory
B	<3	Thin	3–14	Afferent	Autonomic
C	0.2–1.5	None	0.5–2	Afferent	Sensory
					and Motor

*Adapted from Fix and Brueckner (2009)*.

A-group fibers are thick, myelinated, afferent, and efferent, and they also typically have diameters of around 1–22 μm and a conduction velocity of 5–120 m/s. They are typically found in both motor and sensory pathways. B fibers are only moderately myelinated, with diameters = 3 μ m and a conduction velocity ranging from 3 to 15 m/s. C fibers are non-myelinated, and they thus have slower conduction speeds of 2 m/s and thinner diameters of between 0.2 and 1.5 μm.

The cervical branch vagus nerve is made up of about 20% myelinated A and B fibers and 80% unmyelinated C fibers (Vonck et al., 2009). Contrary to earlier studies, which have suggested that C fiber recruitment during VNS was essential for seizure suppression, Kraus et al. (2007) showed that destruction of peripheral C fibers did not influence VNS-induced seizure suppression, and the therapeutic effects of VNS have thus been attributed to the maximal recruitment of thick afferent A and B nerve fibers (Evans et al., 2004). Minimal side effects suggest that stimulation of these fibers is well-tolerated (Helmers et al., 2012).

Similarly, Stefan et al. (2012) showed that tVNS does not elicit painful sensations in the participants, which suggests that afferent C axons and thin myelinated Aδ axons are not activated. A study by Mourdoukoutas et al. (2018) also investigated the fibers that can be activated by tVNS, and they found that at the typically used current of 10 mA, only A-axons and larger B-axons were activated; this is likely due to the diameter of their fibers, implying that C-fibers were too thin to be activated by the applied electrical stimulation.

At the cervical level, the vagus nerve mainly consists of small diameter unmyelinated C fibers (65–80%) and of a smaller portion of intermediate- diameter myelinated B fibers and large-diameter myelinated A fibers. A, B, and C fiber distributions within the carotid vagus nerve have been well-documented (Standring, 2015), enabling the development of computational models to determine the optimal current and pulse width parameters for VNS to activate the myelinated A and B afferent fibers (Helmers et al., 2012). Despite this, the optimal stimulation parameters for VNS are unknown, as the effects of other parameters, such as frequency and duty cycle, are observed post-synaptically in various structures of the brain. Given that these activations cannot be computationally modeled, clinical application and stimulation parameter selection of VNS relies on subjective benefits reported by patients.

In contrast, the distributions of the various nerve fiber types of the ABVN have not been investigated to the level of detail necessary for computational modeling. Therefore, the presence of various nerve fiber types remains speculative and evaluations of intervention efficacy have been based on subjectively experienced therapeutic benefits correlated with other primary and secondary outcomes, such as neuroimaging studies.

As with stimulation of the cervical branches of the vagus nerve with low level electrical currents, stimulation of the ABVN would be expected to activate thick myelinated fibers only and with no activation of the thin diameter unmyelinated C fibers. The ABVN is a general sensory fiber and is one of the few branches to contain no motor fibers. As such, the myelinated fibers found in the ABVN would be expected to be A-group sensory axons rather than B-group autonomic fibers. Only one study has determined the number of myelinated axons that are present in the ABVN (Safi et al., 2016). Around 50% of the myelinated axons were measured to have a diameter of between 2.5 and 4.4 μm, which suggests that they belong to the Aδ group. Nearly 20% of the axons were measured to have a diameter >7 μm, suggesting the fibers belong to the Aβ class. However, the ABVN contains almost six times less Aβ class nerve fibers than those found in the cervical branch of the vagus nerve. This number also varied greatly between individuals, which may explain why some individuals do not experience therapeutic effects after treatment with tVNS, and it may go some way to explain the anatomical basis behind the mechanism and effectiveness of tVNS (Butt et al., 2019).

### 2.3. tVNS for Common Health Conditions

#### 2.3.1. Depression

The mechanism behind the therapeutic anti-depressive effects of VNS and tVNS is still unknown. In 2007, Kraus et al. investigated the acute brain activations of healthy subjects following tVNS through functional magnetic resonance imaging (fMRI), showing hypoactivation of the amygdala, hippocampus, parahippocampal gyrus, and middle and superior temporal gyrus, and hyperactivation in the insula, precentral gyrus, and thalamus (Kraus et al., 2007). These cortical areas are connected both directly and indirectly to the nucleus tractus solitarii (NTS), which receives greatest afferent vagus input. The NTS relays incoming sensory information to the brain via an automatic feedback loop, direct projections to the reticular formation in the medulla, and ascending projections to the amygdala, insula, hypothalamus, thalamus, orbitofrontal cortex, and other limbic regions involved in anxiety and mood regulation via the parabrachial nucleus and the locus coeruleus (Mohr et al., 2011). It is hypothesized that hypoactivation of the amygdala suppresses the hyperactive limbic brain areas, as seen in patients with depression (Mayberg, 1997), through projections from the amygdala to the amygdala–hippocampus–entorhinal cortex of the limbic system (Kraus et al., 2007).

These results are consistent with the acute diminished activity of the limbic system found during VNS (Henry et al., 1998; Chae et al., 2003; Mohr et al., 2011). Interestingly, changes in regional cerebral blood flow induced by VNS are similar to those found in depressed patients treated with selective serotonin reuptake inhibitors (fluoxetine) (Mayberg et al., 2000), either in the amygdala, hippocampus, or parahippocampus (Nemeroff et al., 2006). fMRI studies of patients with depression, following 1 month of tVNS, showed increased functional connections between the default mode network and the precuneus, rostral anterior cingulate cortex, and medial prefrontal cortex. This has also been associated with a reduction in depression severity (Fang et al., 2016) and is similar to results illustrating the therapeutic effects of transcranial magnetic stimulation (Fitzgerald et al., 2006).

Activation of the central nervous system via electrical stimulation of peripheral nerves has become known as the “bottom-up” mechanism, which is a hypothesis based on the neurobiology of the vagus nerve and its effects on neural activity. This is in contrast to the well-known “top-down” mechanism of strategies, such as electroconvulsive therapy and transcranial magnetic stimulation, where the stimulus is applied to central brain structures and subsequently propagates to peripheral sites (Shiozawa et al., 2014). In both human and animal studies, VNS has been shown to elicit changes in neurotransmitters associated with the pathophysiology of depression, including serotonin, norepinephrine, GABA, and glutamate (Ben-Menachem et al., 1995; Krahl et al., 1998; Walker et al., 1999; Dorr and Debonnel, 2006; Manta et al., 2009).

Hein et al. (2012) illustrated the antidepressant effects of 2 weeks of tVNS using an add-on study design, which resulted in significantly improved outcomes on the Beck Depression Inventory (BDI; 27.0–14.0 points). However, no significant changes were observed on the Hamilton Depression Rating Scale (HAMD). Very little information was provided regarding the stimulation parameters that were used; 1.5 Hz unipolar rectangular waves and currents were individually adjusted to maximal but not painful intensities (0–600 mA). In a single blinded clinical trial conducted by Fang et al. (2016) investigating the antidepressant effects of tVNS as a solo treatment, significant improvement was not only seen on the HAMD (28.5–15.0) but also on the Self-Rating Anxiety Scale (SAS; 56.56–42.83) and the Self-Rating Depression Scale (SDS; 66.33–50.56). It is implied that these therapeutic effects may be due to modulation of the resting state functional connectivity of the default mode network, as shown via fMRI imaging. Again, the stimulation parameters used were not comprehensively reported, with density wave adjusted to 20 Hz, a wave width <1 ms, and intensity adjusted based on the tolerance of the patient (4–6 mA).

#### 2.3.2. Epilepsy

In addition to depression, tVNS has also been investigated for its use as a treatment option for drug-resistant epilepsy, a neurological disorder characterized by recurring seizures that affects around 50 million people worldwide (Beghi, 2019). Drug resistance is diagnosed in up to 30% of epilepsy patients (Kwan and Brodie, 2000). Handforth et al. (1998) demonstrated that invasive stimulation of the vagus nerve could suppress the occurrence of seizures and offer a non-pharmacological treatment for epilepsy.

Due to the success of invasive vagus nerve stimulation as a valid treatment option for epilepsy, Stefan et al. (2012) devised a pilot study to investigate whether tVNS would elicit the same anti-convulsive effects. In the pilot study, 10 participants with drug-resistant epilepsy who experienced a minimum of four seizures a month were stimulated on the auricular branch of the vagus nerve transcutaneously through the tragus of the left ear. The stimulation parameters were set to a frequency of 10 Hz with a pulse width of 0.3 ms, and the stimulation intensity was set to the individual's tolerance threshold. The participants were trained to self-administer the tVNS for three 1-h sessions per day as part of their daily routine over a period of 9 months. The participants were encouraged to keep a seizure diary to report the frequency of their seizures both before and during tVNS treatment. In five out of the seven cases that completed the study, the seizure frequency was reduced, which suggested that tVNS could offer seizure-reduction effects.

He et al. (2013) also conducted a pilot study to investigate tVNS as a treatment option for pediatric epilepsy. The stimulation protocol differed to the study of Stefan et al. above, as the stimulation was delivered to the left concha with a frequency of 20 Hz for only 30 min at a time three times daily for 6 months. These parameters were found to also elicit seizure-reduction effects, with a 54% reduction in seizure frequency reported after the 6 months of tVNS treatment. More recently, Liu et al. (2018) found an average seizure reduction of 64.4% in 16 out of 17 of their patients after 6 months of treatment with tVNS. The participants were trained to administer 20 min of tVNS three times a day for 6 months to the left concha with a stimulation frequency of 10 Hz.

The exact mechanism by which tVNS prevents or inhibits seizures is not well-understood. It is thought that afferent projections from the ABVN to the nucleus tractus solitarius (NTS) may be responsible for the anti-convulsive effect, however, the neural networks projecting downstream are unclear (Henry, 2002).

#### 2.3.3. Tinnitus

Tinnitus is the perception of sound in the absence of actual external sound and it affects 10–15% of the general population (Han et al., 2009). Recent imaging studies have suggested that chronic tinnitus is linked to a dysfunction in the auditory system, which results in abnormal neuronal behavior. Pairing of invasive vagus nerve stimulation with sound therapy has been shown to reverse tinnitus in rat models (Engineer et al., 2011), and so Lehtimäki et al. (2013) devised a pilot study to investigate whether tVNS could provide any therapeutic benefits for patients with chronic tinnitus. In addition, they also investigated whether tVNS could affect neuronal activity in the auditory cortex by imaging the brain using magnetoencephalography (MEG).

During the study, 10 participants with chronic tinnitus were stimulated continuously on the left tragus at 25 Hz for 45–60 min over seven sessions. The stimulation was paired with tailored sound therapy, which was classical music with the dominant frequency of the individual's tinnitus removed. After the study, all participants reported improved mood and decreased severity of tinnitus. In addition, MEG scans demonstrated that tVNS modulated the auditory cortical response, which suggests that the auditory system can be accessed and modulated via stimulation of the vagus nerve.

#### 2.3.4. Migraine

A number of studies have looked at applying non-invasive VNS to the neck to treat migraines (Goadsby et al., 2014; Grazzi et al., 2014, 2016; Barbanti et al., 2015; Kinfe et al., 2015b). In all of these studies, the gammaCore device (ElectroCore, 2018) was held against the neck in the region of the cervical branch of the vagus nerve, where two stainless steel electrodes deliver 25 Hz of burst stimulation. Total stimulation time varies between studies, but most give 90 s doses of stimulation at a time. This approach has found success in not only reducing the frequency of migraine attacks in participants but also the severity and resultant disability of the attacks.

In addition to non-invasive VNS at the neck, Straube et al. (2015) also investigated whether tVNS at the tragus would have a similar therapeutic effect on migraine. They devised a study for 46 participants, testing the NEMOS tVNS device applying 25 Hz to the tragus for 4 h per day over 3 months, and they also used 1 Hz to the tragus as an active control. Interestingly, the 1 Hz stimulation elicited a more significant reduction in the number of headache days than the 25 Hz active stimulation. This was an unexpected result and demonstrates that a more robust investigation into different stimulation parameters is crucial.

Again, the mechanism of non-invasive VNS and its effect on migraine is not well-understood. One possibility for the therapeutic effects of non-invasive vagus nerve stimulation is thought to be due to activation of the thalamus, which is responsible for information processing and regulation of cortical activity. In patients with migraine, fMRI studies have shown that there is a decrease in thalamocortical activity, and so stimulation of the vagus may help to counteract this decline (Coppola et al., 2004). Alternatively, it is possible that stimulation of the vagus nerve inhibits nociceptive trigeminal neurons, which may have a pain-inhibitory effect (Randich and Gebhart, 1992).

#### 2.3.5. Pain

Johnson et al. first attempted to study the effect of transcutaneous electrical stimulation of the ear on pain threshold in 1991, with a pilot study of 18 participants receiving low frequency burst stimulation at 2.3 Hz for 15 min on three different auricular sites (Johnson et al., 1991). In this study, pain threshold was noted to increase in 10 out of the 18 participants. Three participants also experienced a prolonged analgesic effect even after the stimulation device was turned off.

This pain-inhibitory effect was also noted by Multon and Schoenen (2005) in a review of clinical data collected from patients with implanted VNS devices. The pain thresholds of the patients and any effect VNS had on headaches was measured and confirmed that implanted VNS offered an analgesic effect. Following on from this review of implanted VNS devices, Laqua et al. (2014) proposed a study to investigate whether non-invasive tVNS could offer the same analgesic effect. Electrical stimulation was delivered for 30 min transcutaneously at the cavum conchae in burst stimulation mode with a changing frequency between 2 and 100 Hz. The individual pain threshold was measured using a Neurometer device that measures the sensory nerve conduction threshold. Of the 21 participants, 15 responded with an increase in pain threshold during tVNS, while six noted a decrease in pain threshold during stimulation. These results, although contradictory, agree with the findings of Johnston et al. and support the view that the analgesic effects of VNS are very much dependent on individual sensitivity alongside stimulation parameters.

Busch et al. (2013) devised a study to investigate whether tVNS has the potential to alter pain processing by examining different submodalities of the somatosensory system. A total of 48 participants were stimulated at the left concha on the inner side of the tragus with a stimulation frequency of 25 Hz. Different tests were devised to measure different pain thresholds, such as heat, mechanical, and pressure-related pain thresholds. The results showed an inhibition of mechanical, heat and pressure pain sensitivity after 1 h of continuous tVNS. Detection thresholds for thermal or mechanical inputs were not altered. These results suggest that tVNS can influence pain processing and offer an inhibitory effect on different pain modalities. Analysis of these different submodalities also suggests that tVNS has an impact on the central pain processing centers rather than just peripheral nociceptor activity.

## 3. Limitations of Current Study Protocols

While the use of tVNS has been shown to elicit therapeutic benefits through various studies (Hein et al., 2012; Lehtimäki et al., 2013; Mei et al., 2014; Straube et al., 2015; Liu et al., 2018), they mostly use different primary and secondary outcome measures and so the comparability between studies is limited. While this is partly due to the application of the technique to various ailments where primary efficacy endpoints differ between studies, there are also major issues with incomplete reporting and inconsistent use of terminology when reporting the results of incomparable and, in some cases, non-reproducible experiments. The stimulation parameters, devices, electrode types and the main findings of relevant studies are summarized in Table 2.

**Table 2:** Summary of previous tVNS clinical trials and studies.

**References**	**Condition/Study**	**Participants**	**tVNS device**	**Electrode type**	**Stimulation Side**	**Stimulation** **Site**	**Sham control**	**Pulse width (ms)**	**Intensity (mA)**	**Freq (Hz)**	**Duty cycle/Time**	**Brain activation**
Keute et al. (2019)	Visual bistable perception	34	Digitimer DS7	Ag/AgCl	L	Cymba Concha	Sham stimulation 25 Hz on ear lobe	0.2 ms	3 mA	25 Hz	30 s on, 30 s off for 40 min	Inferred—tVNS has null effect on dynamics of visual bistable perception; perhaps there is a slight effect of GABA transmission in motor but not in the visual cortex
Zhao et al. (2019)	Post-stroke insomnia	1	NS	NS	L, R	Concha	NS	<1 ms	4-6 mA	20 Hz	30 min twice a day for 4 weeks	Measured—Bold fMRI showed a decrease in functional connectivity between posterior cingulate cortex and other nodes of default mode network but a decrease in functional connectivity between posterior cingulate cortex, lingual gyrus, and cortex surrounding calcarine fissure due to tVNS
Badran et al. (2018b)	Improving oromotor function in newborns	5	Digitimer DS7AH	Custom ear electrode	L	Tragus	NS	0.5 ms	0.1 mA below perception threshold	25 Hz	Max 2 min or less per dose, paired with newborn feeding, stops when newborn stops sucking, up to 30 min a day over 10–22 days	NS
Badran et al. (2018a)	Neuro-physiologic effects of tVNS	17	Digitimer DS7	Ag/AgCl	L	Tragus	Sham stimulation 25 Hz on ear lobe	0.5 ms	200 % of perception threshold	25 Hz	3 × 60 s over 6 min	Measured—Bold fMRI showed active stimulation produced significantly greater increases in the right caudate, bilateral anterior cingulate, cerebellum, left prefrontal cortex, and mid-cingulate than in sham stimulation
Colzato et al. (2018)	Divergent thinking	80	NEMOS, Cerbomed	Titanium*	L	Concha	Sham stimulation 25 Hz on ear lobe	0.2–0.3 ms	0.5 mA	25 Hz	30 s on, 30 s off for 40 min	Inferred—tVNS enhances creativity in selective ways, increased divergent thinking which may be attributed to possible increase in GABA concentration
Fischer et al. (2018)	Conflict-triggered adjustment of cognitive control	21	CM02, Cerbomed	Two titan electrodes	L	Cymba Concha	Sham stimulation 25Hz on ear lobe	0.2–0.3 ms	Below pain threshold (average 1.3 mA)	25 Hz	Continuously for 36 min	Measured—EEG showed tVNS increasing behavioral and electrophysiological markers of conflict adaptation
Jongkees et al. (2018)	Response selection during sequential action	40	CM02, Cerbomed	Two titan electrodes	L	Tragus	Sham stimulation 25 Hz on ear lobe	0.2–0.3 ms	0.5 mA	25 Hz	30 s on, 30 s off for 45 min	Inferred—tVNS improves response selection, possibly due to tVNS increasing GABA concentration, which facilitates action control
Keute et al. (2018)	GABAergic modulation	16	Digitimer DS7	Ambu Neuroline	L	Concha	Sham stimulation 25 Hz on ear lobe	0.2 ms	8 mA (or below pain threshold if not tolerable)	25 Hz	30 s on, 30 s off for 25 min	Measured—EEG demonstrated direct GABAergic effects of tVNS, shows direct effect on electrophysiology after single session of tVNS and suggests non-linear relationship between tVNS and GABA transmission
Liu et al. (2018)	Epilepsy	17	TENS-sm device, Suzhou Medical Audio Supplies	Ear clip	L, R	Cymba Concha and outer ear canal	NS	200 s^†^	4 mA (increased by 2 mA each week until patient could not tolerate or seizures were completely controlled)	10 Hz	3 × 20 min daily for 6 months	Measured—tVNS reduced the number of epileptic seizures and reduced abnormal wave changes shown on electroencephalogram (EEG) monitoring. The EEG changes followed the reduction in the frequency of seizures
Yakunina et al. (2018)	Tinnitus	36	Custom-made	NS	L	Inner tragus and cymba concha	Sham stimulation 25 Hz on ear lobe	0.5 ms	0.1 mA lower than pain threshold	25 Hz	30 s on, 30 s off for 6 × 5 min runs	Measured—fMRI showed tVNS via both the tragus and concha successfully suppressed the auditory, limbic, and other brain areas implicated in the mechanisms involved in the generation/perception of tinnitus via auditory and vagal ascending pathways
Assenza et al. (2017)	Epilepsy	1	NEMOS, Cerbomed	Titanium*	L	External acoustic meatus	Sham stimulation on right ear lobe	NS	Sensitive threshold	NS	4 h	Inferred: tVNS engages same neural fibers as in invasive VNS
Fang et al. (2017)	Depression	38	Suzhou Medical Appliance Factory	Custom ear clip electrodes		Concha	Sham stimulation 20 Hz delivered to superior scapha	0.2 ms	Tolerance threshold (typically between 4 and 6 mA)	20 Hz	Continuously for 30 min twice a day, 5 days a week for 4 weeks	Measured—fMRI shows that tVNS targets left anterior insula, and activation of this region predicts the outcome of treatment for depression
Yu et al. (2017)	Disorders of consciousness	1	NS	NS	L, R	Concha	NS	<1 ms	4–6 mA	20 Hz	30 min twice a day for 4 weeks	Measured—fMRI shows that tVNS activated posterior cingulate/precuneus and thalamus and increased the functional connectivity between posterior cingulate/precuneus and hypothalamus, thalamus, ventral medial prefrontal cortex (vmPFC), superior temporal gyrus, yet decreased the functional connectivity between posterior cingulate/precuneus and the cerebellum
Bauer et al. (2016)	Epilepsy	76	NEMOS, Cerbomed	Titanium*	L	Cymba Concha	Active control 1 Hz stimulation	0.25 ms	Tingling without pain	25 or 1 Hz	30 s on 30 s off for 4 h	NS
Burger et al. (2016)	Fear extinction in health volunteers	38	NEMOS, Cerbomed	Titanium*	L	Cymba Concha	Sham stimulation 25 Hz on ear lobe	NS	0.5 mA	25 Hz	30 s on 30 s off	Inferred—tVNS improved extinction learning, increases in norepinephrine in the prefrontal cortex and limbic areas, such as the amygdala and hippocampus could be a possible working mechanism for the memory enhancing effects of VNS
Cha et al. (2016)	Sudden-onset vertigo	1	ES-420, Ito Company Ltd	Ball electrode	R	Cymba concha, cavum concha, and outer surface of tragus	NS	0.2 ms	Discomfort threshold	30 Hz	4 min each site	Inferred—tVNS may normalize autonomic imbalance due to increased sympathetic response causing vertigo
Frokaer et al. (2016)	Pain threshold	18	NEMOS, Cerbomed	Titanium*	L	Concha	Sham stimulation 30 Hz on ear lobe	0.25 ms	Tingling without pain	30 Hz	60 min	NS
Gaul et al. (2016)	Chronic cluster headache	45	NS	Stainless steel	R	Neck	NS	NS	60 mA	25 Hz	1 ms on, 40 ms off for three doses of 2 min of stimulation twice a day	NS
Grazzi et al. (2016)	Menstrual related migraine	51	gammaCore electroCore LLC	Stainless Steel	L, R	Neck	NS	0.2 ms	Up to 60 mA	25 Hz	Burst (1 ms on, 50 ms off) for 2 min three times a day	NS
Lerman et al. (2016)	Peripheral immune system modulation in healthy humans	20	gammaCore electroCore LLC	Stainless steel	L, R	Neck	Active control 1 Hz stimulation	0.2 ms	Tingling without pain	25 Hz	Burst (1 ms on, 40 ms off) for 2 min	NS
Rong et al. (2016)	Major depressive disorder	160	NS	Ear clips	NS	Concha	Sham stimulation 20 Hz at superior scapha	0.2 ms	Tolerance threshold (typically between 4 and 6 mA)	20 Hz	Continuously for 30 min twice a day	NS
Silberstein et al. (2016a)	Migraine	59	gammaCore electroCore LLC	Stainless steel	R	Neck	Sham device that did not deliver electrical stimulation	NS	Set by the user (up to 60 mA)	NS	2 × 2 min doses delivered 5–10 min apart three times a day	NS
Silberstein et al. (2016b)	Cluster headache	150	gammaCore electroCore LLC	Stainless steel	R	Neck	Sham device delivering 0.1 Hz biphasic pulse	0.2 ms	Set by the user (up to 60 mA)	25 Hz	Burst (1ms on, 40 ms off) for three consecutive 2 min stimulations 1 min apart	Inferred—stimulation of vagus nerve affects hypocretin and orexin pathway that affects pathophysiology of cluster headaches
Trevizol et al. (2016)	Depression	12	Ibramed Neurodyn II	Rubber electrodes	L, R	Mastoid process	NS	0.25 ms	12 mA	120 Hz	30 min a day 10 times over 2 weeks	NS
Fang et al. (2016)	Major depressive disorder	34	NS	Ear clip	L	Concha	Sham stimulation 20 Hz at superior scapha	<1 ms	Tolerance threshold (4–6 mA)	20 Hz	2 × 30 min daily, 5 days a week for 4 weeks	Measured—fMRI showed that after tVNS default mode network functional connectivity showed significant changes in brain regions involved in emotional modulation which is associated with depression severity
Frangos et al. (2015)	Bold fMRI effects of tVNS	12	NEMOS, Cerbomed	Titanium	L	Cymba Concha	Sham stimulation 25 Hz on ear lobe	0.25 ms	Tingling but not painful (0.3–0.8 mA)	25 HZ	Continuously for 14 min	Measured—fMRI shows tVNS significantly affects central projections of the vagus nerve.
Hyvärinen et al. (2015)	Tinnitus	15	Tinnoff Inc	Clip electrode	L	Tragus	Sham stimulation 25 Hz on ear lobe	0.5 ms	Above sensory threshold (~0.5 mA)	25 Hz	Continuously for 6 min	Measured—MEG showed tVNS modulates synchrony of tone-evoked brain activity, especially at the beta and gamma bands
Nesbitt et al. (2015)	Cluster headache	19	gammaCore electroCore LLC	Stainless steel*	L, R	Neck	NS	1 ms	Self-controlled	25 Hz	2 min per dose, up to three doses twice daily	NS
Sellaro et al. (2015b)	Post-error slowing	40	CM02, Cerbomed	Two titan electrodes	L	Outer auditory canal	Sham stimulation 25 Hz on ear lobe	0.2–0.3 ms	0.5 mA	25 Hz	30 s on and 30 s off for 75 min	NS
Sellaro et al. (2015a)	Pro-social behavior	24	CM02, Cerbomed	Two titan electrodes	L	Outer auditory canal	Sham stimulation 25 Hz on ear lobe	0.2–0.3 ms	0.5 mA	25 Hz	30 s on and 30 s off for 26 min	Inferred—tVNS expected to enhance prosocial helping behavior due to activation in the insula and prefrontal cortex but this was not observed
Altavilla et al. (2015)	Migraine	20	gammaCore electroCore LLC	Stainless steel*	NS	Neck	NS	NS	NS	NS	Continuously for 90 s	NS
Barbanti et al. (2015)	Chronic Migraine	50	gammaCore electroCore LLC	Stainless steel*	R	Neck	NS	NS	NS	NS	2 × 120 s doses 3 min apart per migraine	NS
Hasan et al. (2015)	Schizophrenia	20	CM02, Cerbomed	Two titan electrodes	L	Outer auditory canal	No electrical stimulation delivered	0.25 ms	Above perception threshold	25 Hz	30 s on, 180 s off for up to 3 ×3 h a day	NS
Jacobs et al. (2015)	Associative memory in older individuals	30	TENSTem dental, Schwa-medico BV	Circular ear clip	L	External acoustic meatus on inner side of tragus	No electrical stimulation delivered	0.2 ms	5 mA	8 Hz	Twice a day	Inferred—tVNS enhances memory performance by increasing locus coeruleus activity and noradrenalin levels to memory-relevant brain areas.
Kinfe et al. (2015a)	Cluster-Tic syndrome	1	gammaCore electroCore LLC	Stainless steel*	R	Neck	NS	1 ms	12–14 V	25 Hz	Burst for 2 × 90 s doses 15 min apart	NS
Kinfe et al. (2015b)	Migraine and sleep disturbance	20	gammaCore electroCore LLC	Stainless steel*	L, R	Neck	NS	1 ms	0–24 V	25 Hz	Burst for 2 × 2 min twice a day	Inferred—in patients with migraine, and tVNS may help to counteract the decline in thalamocortical activity
Stavrakis et al. (2015)	Atrial fibrillation	40	Grass S88, Natus Neurology Inc	Flat metal clip	R	Tragus	No electrical stimulation delivered	1 ms	Discomfort threshold	20 Hz	Continuously for 60 min following induction of atrial fibrillation	NS
Steenbergen et al. (2015)	Efficiency of action cascading processes in healthy humans	30	CM02, Cerbomed	Two titan electrodes	L	Outer auditory canal	Sham stimulation 25 Hz on ear lobe	0.2–0.3 ms	0.5 mA	25 Hz	30 s on, 30 s off for 45 min	Inferred—tVNS modulates efficiency of action cascading processes, likely via GABA and NE release
Straube et al. (2015)	Migraine	46	NEMOS, Cerbomed	Titanium*	L	Concha	Active control 1 Hz sham stimulation	0.25 ms	Tingling but not painful	1 or 25 Hz	30 s on, 30 s off for 4 h a day for 12 weeks	Inferred—headache decreased more significantly in 1 Hz active control group, possibly due to suppression of nociceptive signaling and pain perception in spinal trigeminal nucleus.tVNS may also alter cortical excitability
Weise et al. (2015)	Parkinson's disease	50	NS	Custom made fine silver wires	L, R	Tragus	NS	0.1 ms	8 mA	0.5 Hz	NS	Measured—scalp electrodes measured activation of brainstem after tVNS and observed somatosensory evoked potentials in the nerve which is believed to reflect neuronal activity
Mei et al. (2014)	Tinnitus	32	TENS-200, Suzhou Medical Supplies Co Ltd	NS	NS	Cavum Concha	NS	1 ms	1 mA	20 Hz	2 × 20 min daily for 8 weeks	NS
Aihua et al. (2014)	Epilepsy	60	TENS-200	NS	L, R	Outer auditory canal and conchal cavity	Sham stimulation 20 Hz on ear lobe	0.2 ms	Individual specific	20 Hz	Continuously for 20 min three times a day	NS
Capone et al. (2015)	Cortical excitability in healthy volunteers	10	Twister, EBM	Ag/AgCl	L	External acoustic meatus at inner side of tragus	Sham stimulation 20 Hz on ear lobe	0.3 ms	8 mA	20 Hz	30 s on, 270 s off for 1 h	Measured—measurement of motor evoked potentials showed a GABA modulation in the motor cortex contralateral to the tVNS stimulation side
Clancy et al. (2014)	Sympathetic nerve activity in healthy humans	48	V-TENS PLUS, Body Clock Health Care Ltd	Modified surface electrodes	NS	Tragus	Disconnected electrodes for sham	0.2 ms	Sensory threshold (10–50 mA)	30 Hz	Continuously for 15 min	NS
Goadsby et al. (2014)	Acute Migraine	30	gammaCore electroCore LLC	Stainless steel*	R	Neck	NS	NS	NS	NS	2 × 90 s doses 15 min apart after migraine onset	NS
Grazzi et al. (2014)	Migraine	30	gammaCore electroCore LLC	Stainless steel*	R	Neck	NS	NS	NS	NS	90 s	NS
Huang et al. (2014)	Impaired glucose tolerance	72	Huatuo TENS-200, Suzhou	NS	NS	Concha	Sham stimulation 20 Hz applied at superior scapha	=1 ms	1.0 (adjusted based on tolerance)	20 Hz	20 min twice daily for 12 weeks	NS
Kreuzer et al. (2014)	Tinnitus	50	Phase I: CM02, Cerbomed Phase II: NEMOS, Cerbomed	Two titan electrodes	NS	NS	NS	NS	0.1–10 mA	25 Hz	Phase I: 30 s on, 180 s off for 6 h per day Phase II: 30 s on, 30 s off for 4 h per day	NS
Laqua et al. (2014)	Pain threshold in healthy humans	22	TNS SM 2 MF, Schwamedico GmbH	Anode: Silver disc Cathode: PECG electrode	L, R	Cavum Concha and Mastoid area	No electrical stimulation delivered	0.2 ms	Perception threshold	2 and 100 Hz	Burst 30 min	Inferred—tVNS produces both anti- and pro-nociceptive effects
Busch et al. (2013)	Pain perception in healthy volunteers	48	STV02, Cerbomed	Bipolar electrode	L	Concha at inner side of tragus	No electrical stimulation delivered	0.25 ms	0.25–10 mA	25 Hz	Continuously for 1 h	Inferred—detailed analysis of different sub modalities of the somatosensory system suggest an impact of t-VNS on central pain processing rather than on peripheral nociceptor activity
He et al. (2013)	Pediatric epilepsy	14	TENS-200	Conductive rubber	L, R	Concha	NS	NS	0.4–1.0 mA depending on tolerance	20 Hz	3 × 30 min a day	Inferred—afferent projections from the ABVN to the nucleus tractus solitarius rather than to the spinal trigeminal nucleus may explain anti-seizure effect
Lehtimäki et al. (2013)	Tinnitus	10	Tinoff pulse generator	Clip electrode	L	Tragus	No electrical stimulation delivered	NS	Above sensory threshold (usually around 0.8 mA)	25 Hz	7 × 45/60 min sessions delivered over 10 days	Measured—MEG shows tVNS can modulate auditory cortical activation
Kraus et al. (2013)	Effects of sham-controlled transcutaneous electrical stimulation	16	Digitimer DS7A	Silver	L	Group I: Anterior wall of ear canal Group II: posterior side of ear canal	Sham stimulation 8 Hz on ear lobe	0.02 ms	Non-painful	8 Hz	4 × 30 s on, 60 s off	Measured—fMRI shows activations and deactivations of certain brain regions, especially frontal and limbic areas depending on area of stimulation, and showed more activation than in sham stimulation
Hein et al. (2012)	Depression	37	Study1: TENS-NET 2000, Auri-Stim Medical Inc Study 2: TENS-NET 1000, Auri-Stim Medical Inc	Headset (4 electrodes placed crosswise)	L, R	Outer auditory canal	No electrical stimulation delivered electrodes unplugged	NS	Study 1: Perception threshold Study 2: 130 μ A	1.5 Hz	Study 1: 1 × 15 min 5 days a week Study 2: 2 × 15 min 5 days a week	NS
Napadow et al. (2012)	Chronic pelvic pain	15	Cefar Acus II, Cefar Medical	Modified press-tack electrode	L	Cymba Concha and slope between antihelix and cavum concha	Sham stimulation 30 Hz on ear lobe	0.45 ms	Strong, non-painful	30 Hz	0.5 s on, matched to respiration for 30 min	NS
Stefan et al. (2012)	Epilepsy	10	NS	NS	L	Tragus	NS	0.3 ms	Tolerance threshold	10 Hz	3 × 1 h a day over 9 months	NS
Schulz-Stübner and Kehl (2011)	Hiccups	1	NMS 300, Xavant Technology	NS	L	Neck	NS	NS	6 mA	1 Hz	30 s	Inferred—Unclear whether hiccups were stopped due to interference with reflex arches at different neuronal levels
Dietrich et al. (2008)	Bold fMRI	4	Cerbomed	Silver	L	Tragus	NS	0.25 ms	4–8 mA	25 Hz	50 s on, 100 s off for 700 s	Measured—Bold fMRI showed tVNS elicited a robust activation in the left locus coeruleus, a brainstem nucleus related to clinical depression as well as bilateral activation of the thalamus
Kraus et al. (2007)	Bold fMRI	22	EMP2 Expert, Schwa-medico GmbH	Silver	L	Tragus	Sham stimulation 8 Hz on ear lobe	0.02 ms	Perception threshold	8 Hz	30 s on, 120 s off three times over 2 days	Measured—fMRI shows tVNS leads to prominent changes in cerebral activation patterns, with marked deactivation in limbic and temporal brain areas
Fallgatter et al. (2003)	Vagus sensory evoked potentials	6	NS	Bipolar electrode	NS	Tragus and acoustic meatus	NS	0.1 ms	8 mA	NS	2 s interstimulus interval	Measured—Evoked potential recordings are far field potentials of post-synaptic brainstem activity from vagus nerve nuclei that can be elicited on electrical stimulation
Johnson et al. (1991)	Pain threshold and autonomic function	24	Microtens 7757	Ag/AgCl and rubber	R	Concha	No electrical stimulation delivered	0.5 ms	Discomfort threshold	2.3 Hz	Burst for 15 min	NS

*NS, not stated. An asterisk indicates that an electrode type was not stated in the study but was assumed by us from the type of the device. A dagger indicates parameters as stated in the original paper but that are outside the normal range (possible typing error)*.

### 3.1. Stimulation Devices

**Figure 4:**
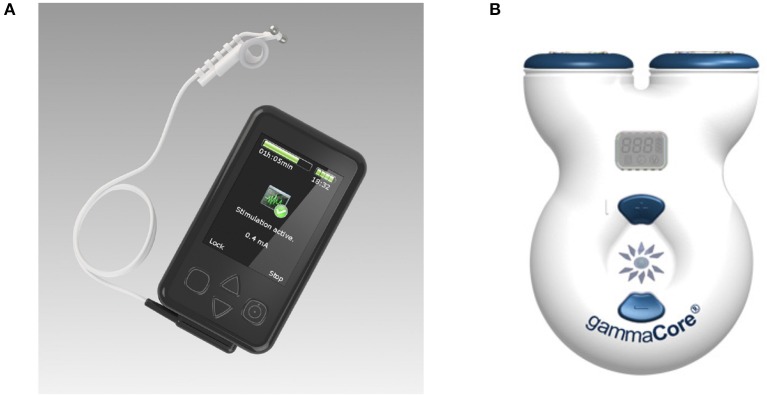
**(A)** Cerbomed NEMOS. Adapted from www.cerbomed.com. **(B)**. Electrocore gammaCore. Adapted from www.gammacore.com.

Research groups generally report the stimulation device used in the experiment, but many of the models used have now been discontinued, and access to their technical specifications is limited. The most commonly used devices are the gammaCore electroCore or Nemos Cerbomed (Figure 4), with a third of the studies included in Table 2 employing them for stimulation (e.g., Grazzi et al., 2014, 2016; Frangos et al., 2015; Straube et al., 2015; Frokaer et al., 2016; Lerman et al., 2016; Silberstein et al., 2016a,b). Almost always, the gammaCore electroCore device is used for stimulation at a neck site (e.g., Goadsby et al., 2014; Grazzi et al., 2014, 2016; Lerman et al., 2016; Silberstein et al., 2016a,b) whilst the NEMOS Cerbomed device is predominantly used for stimulation of the ABVN in the ear. The next most common stimulation device is CM02 Cerbomed, used in Sellaro et al. (2015a,b), Hasan et al. (2015), and Steenbergen et al. (2015) among others. The gammaCore or NEMOS devices are often selected for convenience as they provide an easy-to-use package that includes stimulation electrodes. On the other hand, devices, such as TENS-200 or Digitimer DS7A often require custom-made electrodes. The NMS 300 device from Xavant Technology has also been used (Schulz-Stübner and Kehl, 2011), while the device has not been specified in two studies (Gaul et al., 2016).

#### 3.1.1. ElectroCore Gammacore

The gammaCore, marketed by electroCore, is a handheld tVNS device that stimulates the vagus nerve within the cervical carotid sheath. The device has been granted investigational FDA approval for the acute and/or prophylactic treatment of primary headache and medication overuse headache in adults. Conductive gel is applied to the stimulation surfaces, which are then placed over the sternocleidomastoid muscle. Stimulation intensity is user-controlled (up to 24 V and 60 mA), with individual treatment sessions lasting for 120 s. The treatment can be safely administered multiple times per day; having been applied up to 6–12 times per day in clinical studies (Yuan and Silberstein, 2016b). The remaining stimulation parameters are fixed, delivering 1 ms pulses of 5 kHz sine waves at 25 Hz. It delivers a proprietary pulse waveform that is designed to penetrate through various levels of tissue, including skin, muscle, and nerve sheaths, in order to stimulate the afferent vagus nerve fibers within the carotid sheath. Potential side effects can include tingling under the stimulation electrodes and mild facial twitching at high intensities. It is a limited-use device that is available in two models: 50 doses and 150 doses. Optimal device usage, in terms of the number of stimulations per day and/or total stimulation duration, is yet to be determined.

#### 3.1.2. Cerbomed NEMOS

The NEMOS device (distributed by tVNS Technologies, previously Cerbomed) is a portable transcutaneous electrical nerve stimulator that delivers stimulus to ABVN distributions located in the left cymba concha. NEMOS has been granted the CE mark for the treatment of resistant epilepsy. It is comprised of two main components: the stimulation unit, which houses the battery and pulse generator (and is roughly the size of a mobile phone), and a dedicated ear electrode, which is connected to the stimulator via a cable. Stimulation intensity is user-controlled (up to 25 V), with treatments lasting at least 1 h in three to four sessions per day for a total of 4–5 h. The stimulation current is adjusted until a slight tingling or pulsating sensation is perceived at the stimulation site, implying *Aβ* fiber activation. Prior to stimulation, the user must clean the site of stimulation, as well as the electrodes, to minimize impedance and ensure optimal conductivity. The remaining stimulation parameters are fixed, delivering continuous 0.25-ms-duration monophasic square wave pulses at 25 Hz. Adverse effects may include a slight pain, burning, tingling or itching feeling under the electrode, which dissipates upon electrode removal.

#### 3.1.3. Other

In addition to NEMOS and gammaCore, which are both manufactured specifically for tVNS, stimulation can also be performed by transcutaneous electrical nerve stimulator (TENS) devices, such as TENS-200, V-TENS PLUS, or TENS-NET 2000. Auri-Stim Medical have taken conventional TENS machines, which are typically used in pain management, and repurposed them for stimulating the ear by integrating the electrodes into a headset that can be worn by the user. These devices are portable battery powered control units that can administer tVNS in much the same way as the custom-built units, provided that the electrodes are placed in the correct location in the concha.

The TENS-NET 2000 was approved by the FDA in 2006 and labeled as a nerve stimulator for therapeutic use in depression, anxiety and depression (Hein et al., 2012). User-programmable stimulation parameters include frequency (0.5–100 Hz), intensity (0–6 mA), and mode of stimulation (normal, burst or modulated). However, the polarity of the pulses cannot be varied and are typically monophasic rectangular waves. The stimulation can also be delivered in combination with music or different sounds to enhance the therapeutic effects.

For trials in a clinical or research-based setting, mains-powered medical stimulators, such as Digitimer DS7A or DS5 can be used. These allow complete personalization of stimulation parameters but sacrifice portability. These stimulators are isolated from the mains and can be connected to a computer via BNC cable to allow custom stimulation protocols to be delivered. The Digitimer DS7 is a general-purpose nerve or muscle stimulator for human stimulation and can output up to 100 mA. The frequency and pulse widths of the waves, as well as the duty cycle, are typically programmed on a computer and delivered to the stimulator via BNC cable. There is also the option of alternating the polarity of the pulses, which allows both monophasic and biphasic stimulation pulses to be output.

### 3.2. Electrode Types

Several studies report using gammaCore or NEMOS devices but do not specify stimulation electrode types (e.g., Goadsby et al., 2014; Grazzi et al., 2014; Huang et al., 2014; Altavilla et al., 2015; Barbanti et al., 2015; Nesbitt et al., 2015; Straube et al., 2015). In these cases, we assume that stimulation electrodes provided with the device were not modified for the study, and we report manufacture specifications for the gammaCore/NEMOS electrodes in Table 2 (noted with an asterisk).

When reported, the most commonly used stimulation electrodes are made of titanium (for the ear) (Hasan et al., 2015; Sellaro et al., 2015a,b; Fischer et al., 2018; Jongkees et al., 2018) or stainless silver (for the neck) (Kinfe et al., 2015b; Gaul et al., 2016; Grazzi et al., 2016; Lerman et al., 2016; Silberstein et al., 2016a,b). Silver is also used as an electrode material for stimulation of ABVN (e.g., Laqua et al., 2014; Capone et al., 2015; Weise et al., 2015; Badran et al., 2018a; Keute et al., 2019). Information about stimulation electrodes is often somewhat insufficient: the material or size of the electrodes are often not specified (Stefan et al., 2012; Hyvärinen et al., 2015; Weise et al., 2015; Fang et al., 2016; Yakunina et al., 2018). This limits our collective understanding of the electrode-tissue interface and its interactions. However, the fact that the patient-specific pain threshold is often set as the stimulation current provides some control for variations in the electrode-tissue impedance.

### 3.3. Stimulation Site

**Figure 5:**
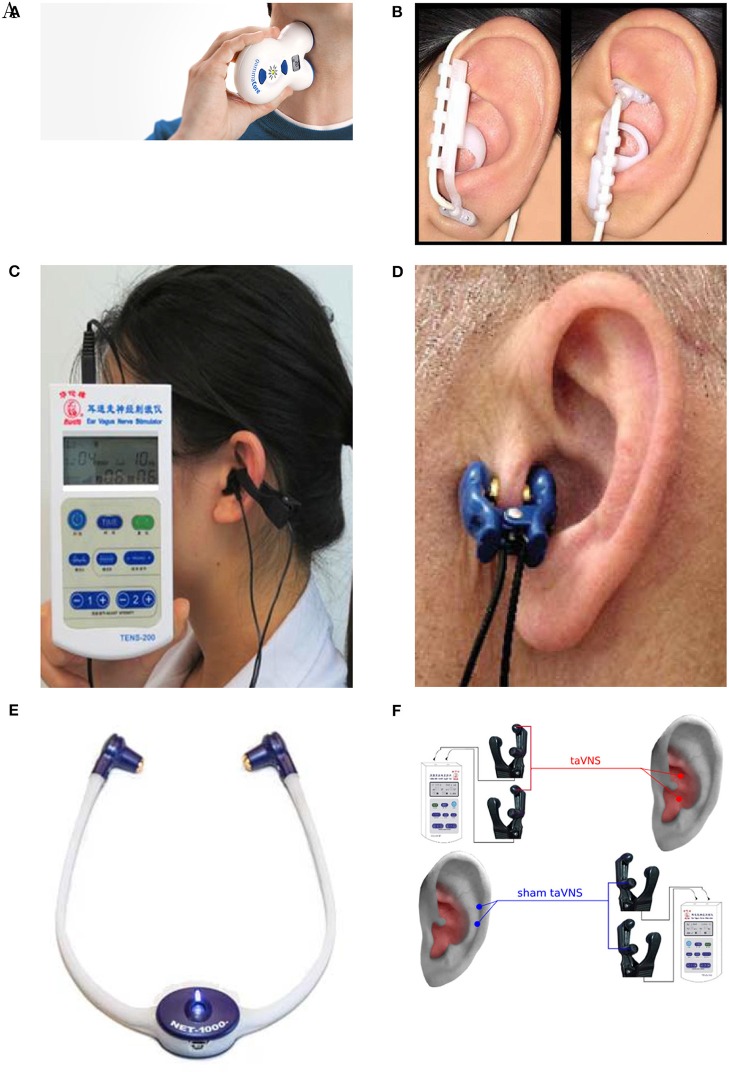
Stimulation electrode positions. **(A)** Neck stimulation using a gammaCore device (Silberstein et al., 2016b). Image courtesy of electroCore Inc, electrocore.com. **(B)** Earlobe sham and cymba concha stimulation using NEMOS electrodes (Frangos et al., 2015). **(C)** External ear canal and concha stimulation using a TENS device from Suzhou (Liu et al., 2018). **(D)** Tragus stimulation (Lehtimäki et al., 2013). **(E)** External ear canal stimulation using a headset NET-1000 (Hein et al., 2012). Image courtesy of Auri-Stim Medical Inc, net1device.com. **(F)** Concha and cymba concha active stimulation (Rong et al., 2016). All figures reproduced with permission.

Out of 61 studies included in Table 2, 13 use the neck as a stimulation location (Figure 5A) (see Gaul et al., 2016; Grazzi et al., 2016; Lerman et al., 2016; Silberstein et al., 2016a,b among others). Discrepancies exist between reported stimulation locations within the studies that stimulate ABVN (Figures 5B–F). This is true even when the same device is used; for example, Straube et al. (2015) and Frangos et al. (2015) both use the NEMOS device, yet report the concha and cymba concha as the location of stimulation, respectively. The stimulation location is often dictated by the geometry of an electrode, with clip electrodes typically attached to tragus or concha (Figures 5C,D) (Lehtimäki et al., 2013; Mei et al., 2014; Straube et al., 2015; Fang et al., 2016; Rong et al., 2016; Liu et al., 2018). Often the outer audio canal is reported as a site for stimulation, without further clarification for the location of an electrode (Hasan et al., 2015; Sellaro et al., 2015a,b; Steenbergen et al., 2015). Given that studies have been done in different participant groups with different clinical conditions and with different stimulation parameters, it is difficult to conclude an optimal stimulation site for any particular disorder.

Initial investigations in this direction have been undertaken in Napadow et al. (2012) and Kraus et al. (2013). Napadow et al. concluded that the concha is the best site for stimulation, while Kraus et al. proposed that the anterior wall of the ear canal is the best for efficacy and participant's convenience. Studies, such as these are progressing in the right direction, but a more systematic approach is required to investigate the effect of the electrode placement on the ABVN recruitment and corresponding neural activations.

Although research groups acknowledge that the ABVN innervates the tragus, concha, and cymba concha as per Peuker and Filler's anatomical studies (Peuker and Filler, 2002), most do not mention antihelix innervation. Selection of the stimulation site appears to be arbitrary, either predetermined by the device employed in the experiment or based on other previous studies without providing any evidence or explanation for the designated stimulation site.

### 3.4. Stimulation Waveform

Most studies employ monophasic rectangular waveforms often set by the specifications of the device used (Hein et al., 2012; Busch et al., 2013; Stavrakis et al., 2015; Badran et al., 2018a; Yakunina et al., 2018), while some others report using biphasic waveform stimulation (Stefan et al., 2012; Hyvärinen et al., 2015; Liu et al., 2018). Lerman et al. (2016) and Silberstein et al. (2016b) reported using sinusoidal wave bursts; however, it is not clear from these studies whether this waveform is more optimal to activate neural fibers. The use of devices that employ “proprietary” or “modified” waveforms, such as electroCore's gammaCore, further hinders insights into the effect of stimulation waveforms on key research outcomes.

### 3.5. Stimulation Intensity

The justifications mentioned above are also employed to motivate the choice of stimulation parameters. Some studies have credited (Kraus et al., 2007; Polak et al., 2009) as having defined the optimal stimulation parameters for tVNS. However, further investigation suggests that these studies only elucidate the optimal stimulus intensity to induce the greatest vagus sensory evoked potential (VSEP) amplitudes (Polak et al., 2009), and that tVNS causes hypo- and hyperactivations of brain regions of interest relating to a decrease in depressive symptoms (Kraus et al., 2007). As Polak et al. (2009) have stated, “we chose a stimulation intensity of 8 mA allowing detection of sufficient VSEP amplitudes without perception of pain,” which reveals nothing about the effects observed post-synaptically in various structures of the brain.

They also acknowledge that VSEP amplitudes are directly correlated to stimulation intensity (i.e., stimulation intensities >8 mA would elicit even greater VSEP amplitudes). Similarly, the studies of Kraus et al. (2007) showed no systematic effects of stimulation parameters on brain activation, although they did illustrate that tVNS does indeed elicit acute changes in brain regions that are related to a decrease in depressive symptoms similar to those caused by VNS. Therefore, neither of these studies can claim to have identified the optimal stimulation parameters of tVNS for the greatest decrease in depressive symptoms or seizure occurrence.

Furthermore, despite electrical current values being reported, the amount, or amplitude, of energy delivered to tissues is largely unknown given the substantial effect of electrode and tissue impedance and need for precise placement (e.g., a stated current of 8 mA presupposes that there is no impact of tissue impedance variation, and therefore voltage, and also neglects waveform shape, rise/fall-time, or any resultant residual charge). The stimulation current is often set according to the subject's sensitivity or just below pain threshold (Napadow et al., 2012; Frangos et al., 2015; Cha et al., 2016; Lerman et al., 2016; Fischer et al., 2018; Yakunina et al., 2018). Given the different stimulation tolerance of different participants, stimulation amplitudes vary over a wide range (from 0.5 mA in Jongkees et al., 2018 to 12 mA in Trevizol et al., 2016). Undoubtedly, the stimulation electrode electrochemistry also contributes to the maximum current that is tolerated by a participant.

### 3.6. Stimulation Frequency

With regard to stimulation frequency, the currently used range of 20–30 Hz has never been validated for its therapeutic effects (Laqua et al., 2014). Following studies showing that stimulation frequencies of 50 Hz and above can cause major and irreversible damage to the vagus nerve during VNS (Agnew and McCreery, 1990), stimulation frequencies between 20 and 30 Hz were arbitrarily selected in order to limit adverse events associated with direct stimulation of the carotid sheath and were subsequently approved by the FDA (Groves and Brown, 2005). Lower frequencies of stimulation have also been explored. Liu et al. (2018) have found that 10 Hz tVNS for 20 min periods three times per day for 6 months reduced the number of seizures, while 8 Hz stimulation leads to activation in frontal and limbic brain areas as measured by fMRI (Kraus et al., 2007). Straube et al. (2015) have seen a stronger reduction in migraine episodes when stimulating at 1 Hz than when stimulating at 25 Hz. Thus, it should not be assumed that stimulation frequencies within the 20–30 Hz range are optimal for tVNS, and additional controlled studies are warranted to elucidate the effect of stimulation frequency rather than a selection based on past FDA approval of a related, yet different, technique.

## 4. Brain Activation

Several studies have speculated about the brain areas that are activated as a result of tVNS (Schulz-Stübner and Kehl, 2011; Busch et al., 2013; Laqua et al., 2014; Colzato et al., 2018; Jongkees et al., 2018). For example, Burger and Verkuil (2018) proposed that tVNS leads to activation in limbic areas, such as the amygdala and hippocampus, whereas Cha et al. (2016) suggested that it normalizes autonomic imbalance due to an increase in sympathetic response in patients with vertigo. In contrast, Silberstein et al. (2016b) proposed that stimulation of the vagus nerve affects hypocretin and orexin pathways in people with cluster headache, while Kinfe et al. (2015b) hypothesized that tVNS may help counteract the decline in thalamocortical activity in people with migraine and sleep disturbances. Jacobs et al. (2015) suggested that tVNS enhances memory performance by increasing neural activity in the locus coeruleus. It is clear that researchers have proposed different effects of tVNS on neural activation depending on the focus of their study. Measuring neural activity using techniques, such as fMRI, EEG, or MEG is critically important to confirm proposed hypotheses.

Brain activation in response to tVNS has been measured in Kraus et al. (2007), Kraus et al. (2013), Dietrich et al. (2008), Lehtimäki et al. (2013), Capone et al. (2015), Frangos et al. (2015), Hyvärinen et al. (2015), Weise et al. (2015), Fang et al. (2016), Yuan and Silberstein (2016b), Yu et al. (2017), Badran et al. (2018a), Fischer et al. (2018), Liu et al. (2018), Yakunina et al. (2018), Keute et al. (2019), Zhao et al. (2019), and Fallgatter et al. (2003). Most of these studies have been conducted in the last 5 years, with the exception of three that pioneered this field in the 2000s (Fallgatter et al., 2003; Kraus et al., 2007; Dietrich et al., 2008). Dietrich et al. (2008) showed that tVNS elicits activation in the left locus coeruleus, a brainstem nucleus that is implicated in clinical depression, as well as bilateral activation in the thalamus. Fallgatter et al. (2003) measured evoked potentials of post-synaptic brainstem activity from vagus nerve nuclei that can be elicited by electrical stimulation. Using fMRI, Kraus et al. (2007) demonstrated that tVNS leads to prominent changes in cerebral activation with marked deactivation in limbic and temporal brain areas.

Later fMRI studies have shown that active tVNS (i) produces a significantly larger increase in neural activity in the right caudate, bilateral anterior, left prefrontal cortex, cerebellum, and mid-cingulate than sham stimulation (Badran et al., 2018a); (ii) leads to a decrease in functional connectivity between posterior cingulate cortex and lingual gyrus (Zhao et al., 2019); and (iii) suppresses the auditory, limbic, and other brain areas implicated in the mechanisms involved in the generation of tinnitus (Yakunina et al., 2018).

EEG studies have shown a direct effect of tVNS on electrophysiological markers of conflict adaptation (Fischer et al., 2018) and on the number of seizures (Liu et al., 2018). MEG recordings have shown that tVNS modulates synchrony of tone-evoked brain activity, especially in the beta and gamma bands (Hyvärinen et al., 2015).

It is not clear why the areas of brain activation vary between these studies, but it may be due to the different conditions presented by the participants. Due to the variation in results, different studies have proposed different underlying mechanisms for tVNS, and, as such, there can be no clear conclusions made from the different imaging studies. Despite the breadth of research being undertaken, many questions remain regarding the most effective stimulation sites and parameters. As many of the described methods differ in the parameters and protocols applied, there is currently no firm evidence on the optimal parameters to provide the greatest benefit to subjects.

### 4.1. Side Effects

Although tVNS is on the whole well-tolerated as a treatment option, a number of different mild side effects have been noted, which Redgrave et al. (2018) summarized in their review. Common side effects include tingling or pain around the stimulation site, with some participants reporting itching or redness (Busch et al., 2013; He et al., 2013; Goadsby et al., 2014; Kreuzer et al., 2014; Rong et al., 2014; Barbanti et al., 2015; Hasan et al., 2015; Jacobs et al., 2015; Kinfe et al., 2015b; Stavrakis et al., 2015; Straube et al., 2015; Weise et al., 2015; Bauer et al., 2016; Cha et al., 2016; Grazzi et al., 2016; Lerman et al., 2016; Silberstein et al., 2016a,b; Trevizol et al., 2016). Other less common side effects that have been observed in <1% of the study population include gastrointestinal issues, such as nausea or vomiting (Schulz-Stübner and Kehl, 2011; Kreuzer et al., 2014; Jacobs et al., 2015; Bauer et al., 2016; Silberstein et al., 2016b; Trevizol et al., 2016), headache (Stefan et al., 2012; Kreuzer et al., 2014; Jacobs et al., 2015; Bauer et al., 2016; Gaul et al., 2016; Lerman et al., 2016; Silberstein et al., 2016a; Trevizol et al., 2016), heart palpitations (Bauer et al., 2016), facial drooping (Goadsby et al., 2014; Silberstein et al., 2016b), dizziness (Aihua et al., 2014; Goadsby et al., 2014; Huang et al., 2014; Kreuzer et al., 2014; Rong et al., 2014; Jacobs et al., 2015; Bauer et al., 2016; Gaul et al., 2016), vocal hoarseness (Stefan et al., 2012; Goadsby et al., 2014; Kreuzer et al., 2014), and nasopharyingitis (Bauer et al., 2016; Gaul et al., 2016). There is currently no study that links stimulation parameters or dose to the rate of side effects experienced, which should be a priority for future research in the field, and clear reporting of both side effects and stimulation parameters is important to be able to observe any trends.

## 5. Discussion and Future Directions

This review has focused on a mechanistic understanding of transcutaneous vagus nerve stimulation (tVNS), with a detailed discussion of stimulation parameters, sites of stimulation, and devices used in current research. It should be noted that there is an ongoing discussion about the translation of non-invasive neural stimulation therapies into clinical practice. Transcranial magnetic stimulation (TMS) is another type of non-invasive neural stimulation therapy that is becoming more commonly used as a treatment option for different conditions, although use of the device is limited to clinical settings where it is operated by a healthcare professional. In contrast, transcranial direct stimulation (tDCS) (Wexler, 2015), much like tVNS, is a portable treatment option that does not require operation by a professional.

On the one hand, the affordability and easy availability of these devices, and an absence of severe adverse events, has led to a “do-it-yourself” movement that uses tDCS and tVNS at home for self-improvement purposes. Researchers are still trying to understand the risks and benefits of these techniques and fear that uncontrolled use may lead to unintended consequences (Bikson et al., 2013).

The situation is further complicated by the fact that, for regulatory purposes, the definition of a medical device focuses on the intended use of a device rather than the mechanism of action. This implies that manufacturers can skirt regulation by careful wording about the intended use. However, it is clear that a thorough risk analysis requires a sound understanding of the mechanism of action. Therefore, to promote the safe and efficacious use of tVNS in future, it is important to understand the mechanism of action of this promising technique.

The actual mechanisms of tVNS are still poorly understood. Many studies contradict the findings of similar studies and there is often very little homogeneity in results, making it difficult to draw conclusions from the findings. It has been proven by a number of studies that tVNS affects the same neural pathway as invasive VNS (He et al., 2009; Van Leusden et al., 2015); however, there is no conclusive evidence to explain why tVNS elicits therapeutic effects. It is therefore important for future studies to focus on the mechanism of action by following rigorous protocols that include objective measures of brain activation. It is also important that past assumptions about the effects of tVNS on brain neural activation and function do not restrict the direction of future investigations.

Given that stimulation parameters vary significantly between studies, a systematic approach is required to identify the optimal stimulation intensity, pulse width, waveform and frequency that provides the greatest clinical benefit. This may require participant-specific adjustment of parameters in a closed-loop setup, where stimulation parameters are set online, based on recorded neural activity. All current stimulation strategies for tVNS devices rely on open-loop control of the stimulation parameters, where the levels are set at the beginning of the stimulation protocol and do not change in response to any continuous measurement of the level of neuronal activation. It is reasonable to expect different outcomes in response to open-loop electrical stimulation between participants and between trials due to different ongoing brain activities at the time of stimulation. While many studies have been successful in using open-loop techniques (Barbanti et al., 2015; Trevizol et al., 2016; Liu et al., 2018), the outcomes differ from patient to patient. A customized closed-loop controller will allow the manipulation of specific patient-based neural responses. Pioneering steps in closed-loop VNS have been reported in Boon et al. (2015) and Fisher et al. (2016).

A closed-loop protocol will require continuous measurements of behavioral outcomes or brain activity. Since behavioral measures are often imprecise, it is preferable that imaging techniques, such as EEG or MEG, be used during the stimulation protocol to study neural activation and information transfer. The EEG signal has low spatial resolution that makes it difficult to interpret brain network connectivity. In contrast, MEG imaging has higher spatial resolution than EEG and higher temporal resolution than fMRI. The reconstruction of neuronal activity sources from MEG has less sensitivity to model approximations and smaller localization errors than EEG reconstruction. The MEG is sensitive to a wide range of frequencies in the oscillatory brain signals and has full brain coverage. There exist various techniques to reconstruct the anatomical origin of brain activity from MEG signal. When a structural MRI scan is available, it is possible to coregister MEG signals to anatomical locations. These advantages of MEG offer a powerful tool to study connectivity between brain areas and analyze brain networks and function (Baillet, 2017).

Such combined neuroimaging techniques can also help to resolve the origin of vagus connections in the brain. The “vagus” in the term tVNS is based on the assumption that the auricular branch of the vagus nerve has been activated. Some researchers believe that the auricular branch of the vagus is a misplaced branch of the trigeminal nerve and carries somatic-not visceral-afferent fibers. In this respect, this nerve is just like the trigeminal nerve branches to the rest of the face. If this hypothesis is true, then the auricular nerve would not connect to the NTS in the brain but rather to the trigeminal-or possibly paratrigeminal-nuclei. The latter nucleus receives cough receptor afferents from the airways, which may be why the auricular branch (“Arnold's nerve”) can stimulate coughing (Gupta et al., 1986). However, a recent investigation of central neuronal projections from nerves innervating the external auricle in rats, appears to challenge an opinion that stimulation of the tragal nerve is conducted by the auricular branch of the vagus (Mahadi et al., 2019). Similar studies need to be done in primates to confirm whether the same conclusion may apply to humans.

Many studies have very few participants, with some having as few as one. This leads to difficulties in concluding whether the results or proposed mechanisms can be generalized to a larger population. To avoid the risk of accidentally having extreme or biased results, studies with a large number of participants are required. Due to heterogeneous populations with various health conditions and different medications and treatment responses often enlisted for a study, it is impossible to generalize to another condition or to a healthy group. Rigorous studies with a large number of healthy participants, where a wide range of stimulation parameters are tested within a participant and between a cohort, are needed to draw solid, evidence-based conclusions. Such studies may also reveal biomarkers for responders and non-responders to tVNS.

There has been very little investigation into how long the effects of tVNS last after the stimulation period has ended. Most clinical trials involve daily stimulation periods over the course of the trial, with the therapeutic results measured concurrently. Studies, such as that of Hein et al. (2012), have compared therapeutic results after the 2-weeks treatment period of daily stimulation to the baseline results recorded from before the stimulation period. Other studies (Huang et al., 2014; Mei et al., 2014; Rong et al., 2016) measured the therapeutic effects of daily stimulation continuously over set intervals during the trial period. Many studies found that participants who completed the entire treatment study had a better response to tVNS than those who dropped out, and longer treatment periods corresponded with better therapeutic outcomes (He et al., 2013; Bauer et al., 2016; Silberstein et al., 2016a; Liu et al., 2018). However, these studies did not offer a follow-up to see whether the effects of tVNS were long-lasting or remained after the cessation of the treatment period. In the case study by Zhao et al. (2019) on a single participant with insomnia, after 2 weeks of twice daily tVNS the treatment was stopped, but the participant still felt an improvement at the follow-up meeting, 3 months after the trial period. Similarly, Trevizol et al. (2016) had a stimulation period of 10 days, but found the clinical response remained stable 1 month after stimulation had stopped.

Some studies into the pain-relieving effects of tVNS have investigated whether the effects last for some time after the stimulation. Johnson et al. (1991) and Napadow et al. (2012) reported that an analgesic effect was present for up to 15 min after stimulation ceased. Other studies measured the therapeutic effects immediately after stimulation (Capone et al., 2015; Stavrakis et al., 2015; Keute et al., 2019) or at the same time as stimulation (Fallgatter et al., 2003; Kraus et al., 2007, 2013; Dietrich et al., 2008; Lehtimäki et al., 2013). This may offer interesting results for the measurement of brain activity as a result of tVNS but does not indicate whether these effects are long-lasting. Indeed, Frangos et al. (2015) noted that neural activation gradually returned to the baseline after tVNS was stopped. Immediate measurement of the therapeutic effects of tVNS do not therefore suggest whether these effects are merely a temporary result of stimulation or long-lasting.

When long periods of stimulation are required to achieve the maximum effect, it is unreasonable to expect participants to attend prolonged sessions several times per day. Therefore, portable stimulators are required, but gammaCore and NEMOS are currently the only tVNS devices available. It is difficult to track participants' compliance with these devices and record how stimulation parameters change over time. More research is required to produce miniaturized devices that are convenient and safe to use.

## 6. Conclusion

tVNS has proven to be an effective way to modulate the central nervous system in some cases. However, the mechanism of action is not clear, and the robustness of the results is yet to be proven. The technique is safe and convenient with only a few relatively minor side effects reported. More rigorous systematic studies are required to investigate the effects of stimulation parameters, sites of stimulation, and electrode types on brain activation and clinical outcomes. Current limitations in study protocols may lead to difficulties in obtaining regulatory approval and challenges in translating research studies into clinical practice.

## Author Contributions

JY and PS conceived and designed the idea. JY, CK, TK, and PS wrote the manuscript. All authors aided in interpreting the results and commented on the manuscript.

## Conflict of Interest

The authors declare that the research was conducted in the absence of any commercial or financial relationships that could be construed as a potential conflict of interest. The Handling Editor declared a past co-authorship with one of the authors TK.
